# Supplementary material for: C5aR1 antagonism suppresses inflammatory glial responses and alters cellular signaling in an Alzheimer’s disease mouse model
Source: Nat Commun. 2024 Aug 15;15:7028. doi: 10.1038/s41467-024-51163-6 (PMC11327341; doi:10.1038/s41467-024-51163-6)
Supplement: Supplementary file 1 — Supplementary Information [file 41467_2024_51163_MOESM1_ESM.pdf]

C5aR1 antagonism suppresses inflammatory glial responses and alters cellular signaling in Alzheimer's disease mouse model

Authors: Nicole D. Schartz[1], Heidi Y. Liang[2], Klebea Carvalho[2], Shu-Hui Chu[1], Adrian Mendoza-Arvilla[1], Tiffany J. Petrisko[1], Angela Gomez-Arboledas[1], Ali Mortazavi[2], Andrea J. Tenner[1,3,4]*

Supplementary Figures

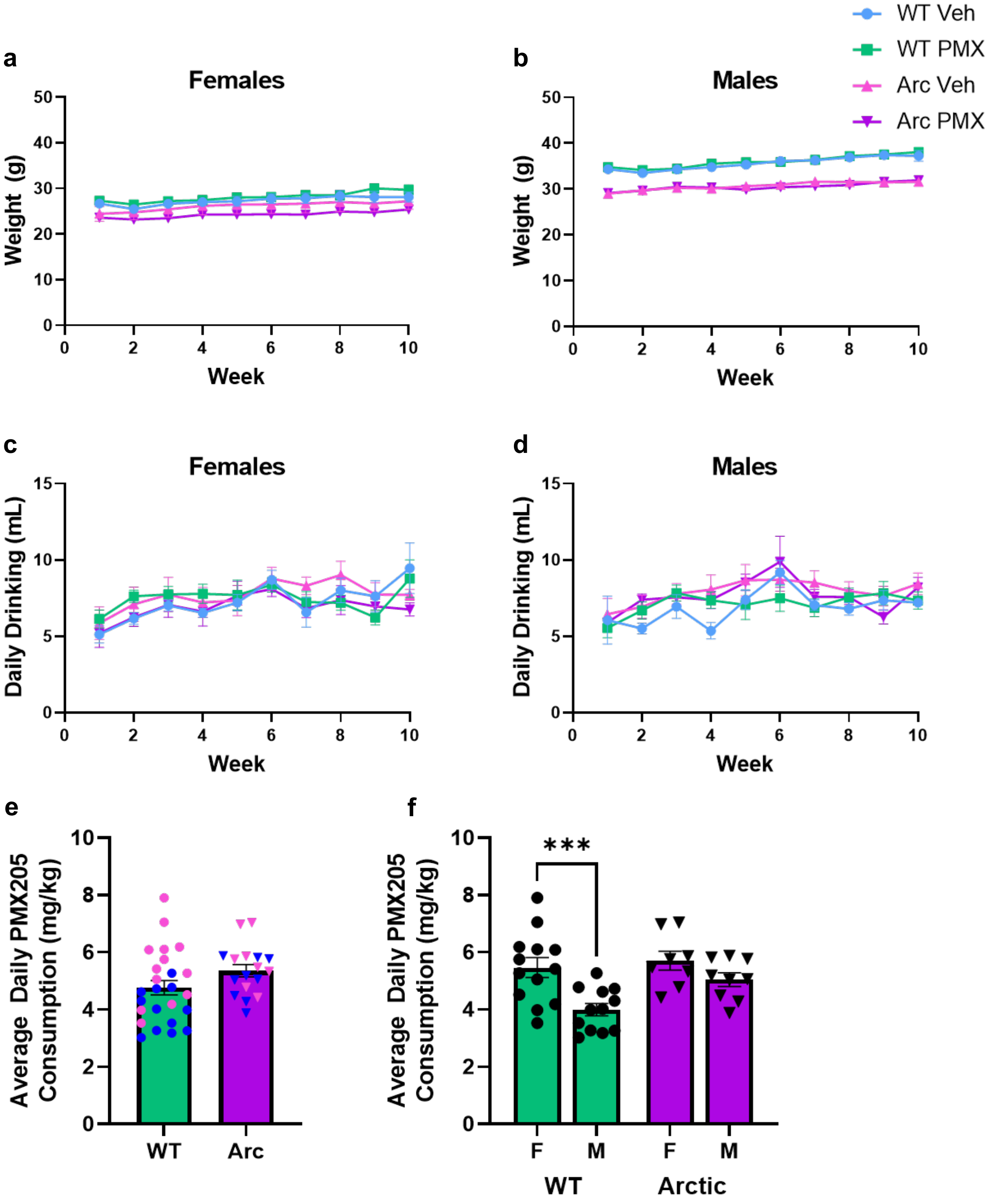

Figure S1: PMX205 consumption did not have toxic physiological effects. (**a-b**) Weight log (weekly) of female (**a**) and male (**b**) mice through10 weeks of treatment with or without PMX205 (**c-d**) Average volume of water consumed per week throughout treatment. (**e**) PMX205 dose was calculated based on volume consumed by each animal (females shown in pink points, males in blue). (**f**) Comparison of average daily PMX205 dose between males and females. WT males had a 26.8% smaller dose than females. Although there was no difference in volume consumed, the relative dose in males was smaller due to higher body weight. Data shown as mean ± SEM. *** $p < 0.001$, Two-way ANOVA with Sidak's *post hoc* test. N= 9F12M (WT-H_2O), 12F12M (WT-PMX), 8F9M (Arc-H_2O), 8F9M (Arc-PMX).

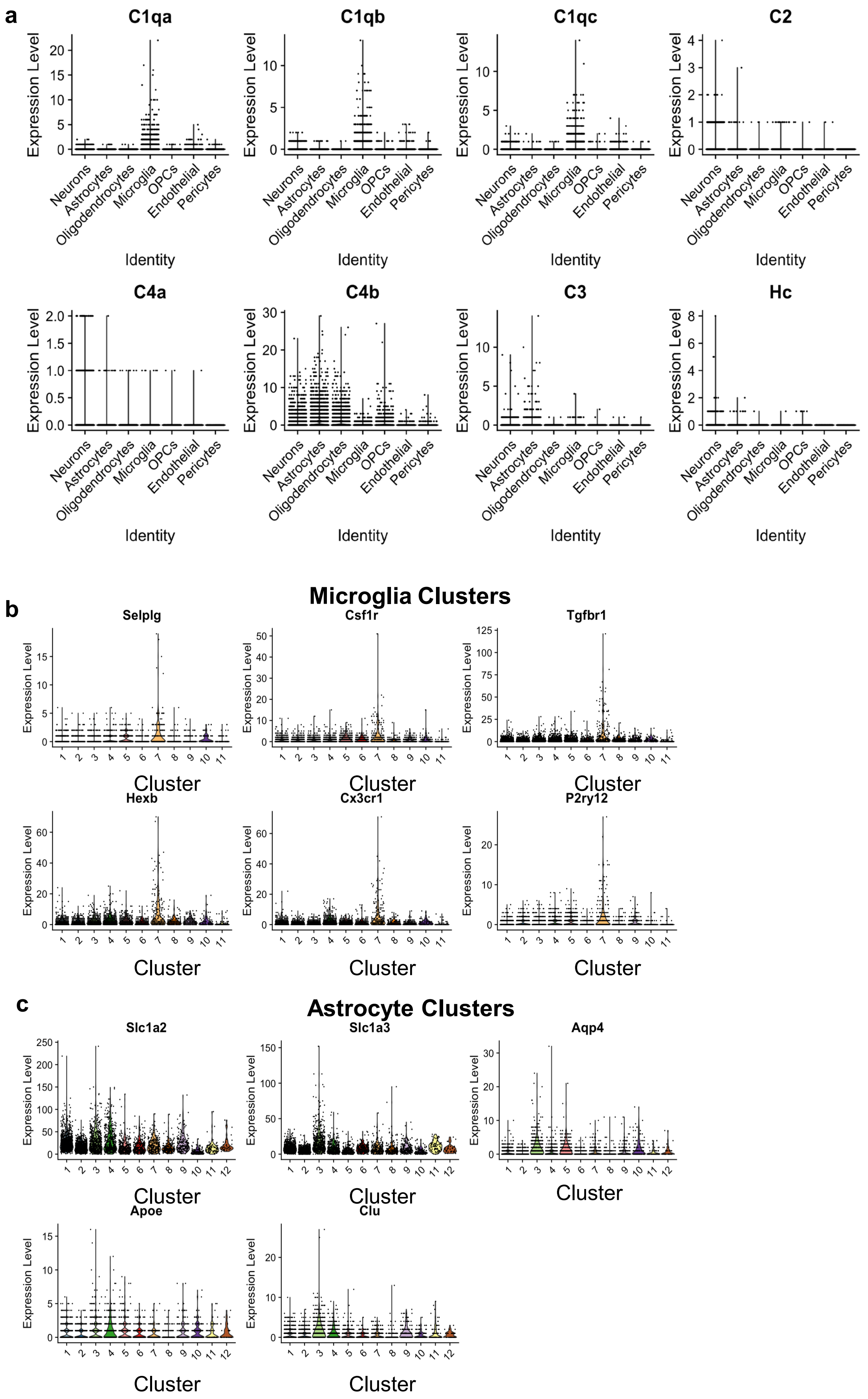

Figure S2: Cell-specific complement expression and validation of microglia and astrocyte clusters with expression of known genes. (**a**) Expression level (count) of complement genes in different cell populations (**b**) Expression levels of microglial genes Selplg, CSF1r, Tgfbr1, Hexb, Cx3cr1, P2ry12 in each microglia cluster. (**c**) Expression levels of astrocytic genes Slc1a2, Slc1a3, Aqp4, Apoe, and Clu in each astrocyte cluster.

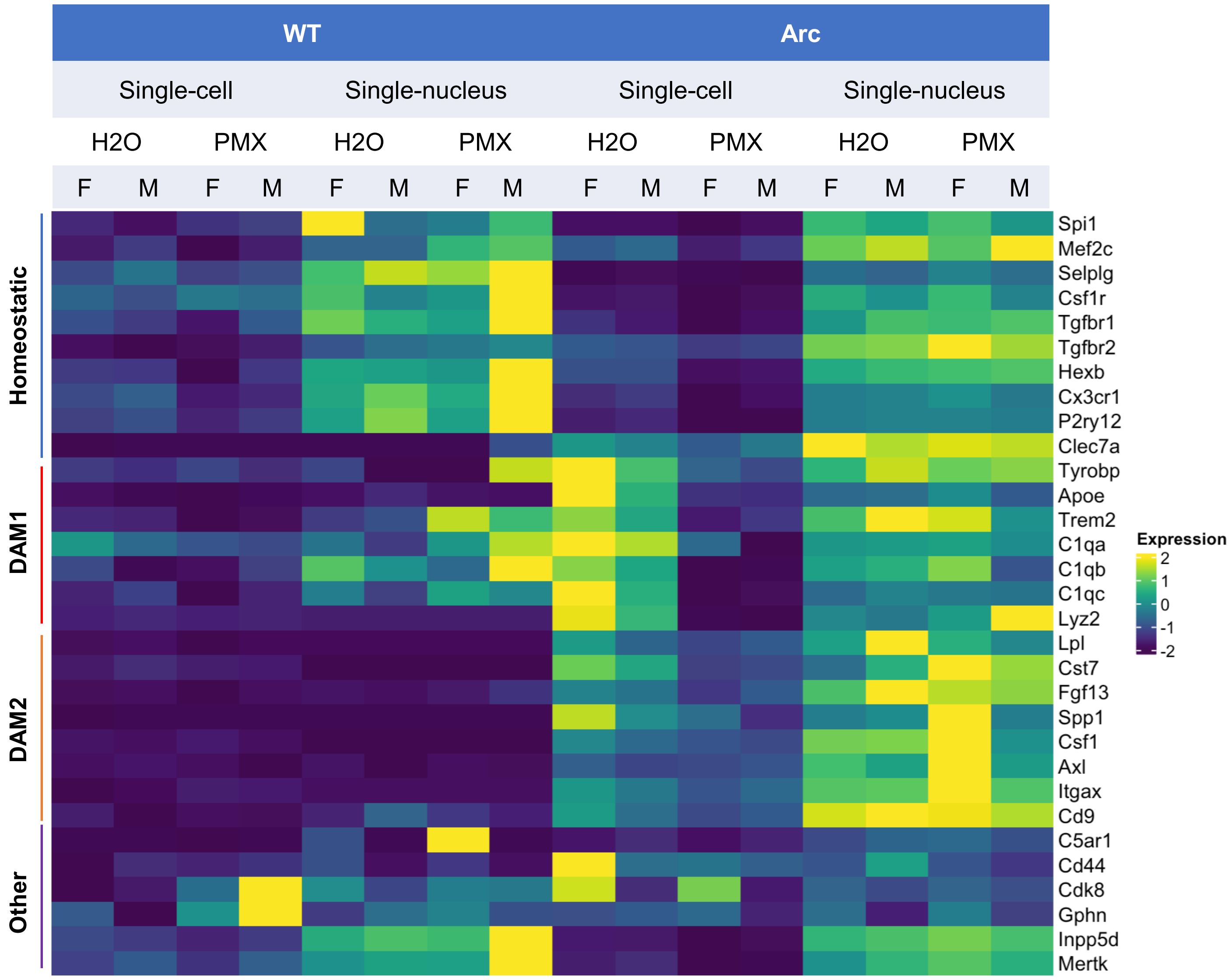

Figure S3: Comparison of expression of microglial genes in single-cell and single nucleus and by sex. Relative expression of homeostatic, DAM1, and DAM2 genes was visualized in single-cell and single-nucleus-derived samples. Expression was assessed by genotype, treatment of PMX205, and sex.

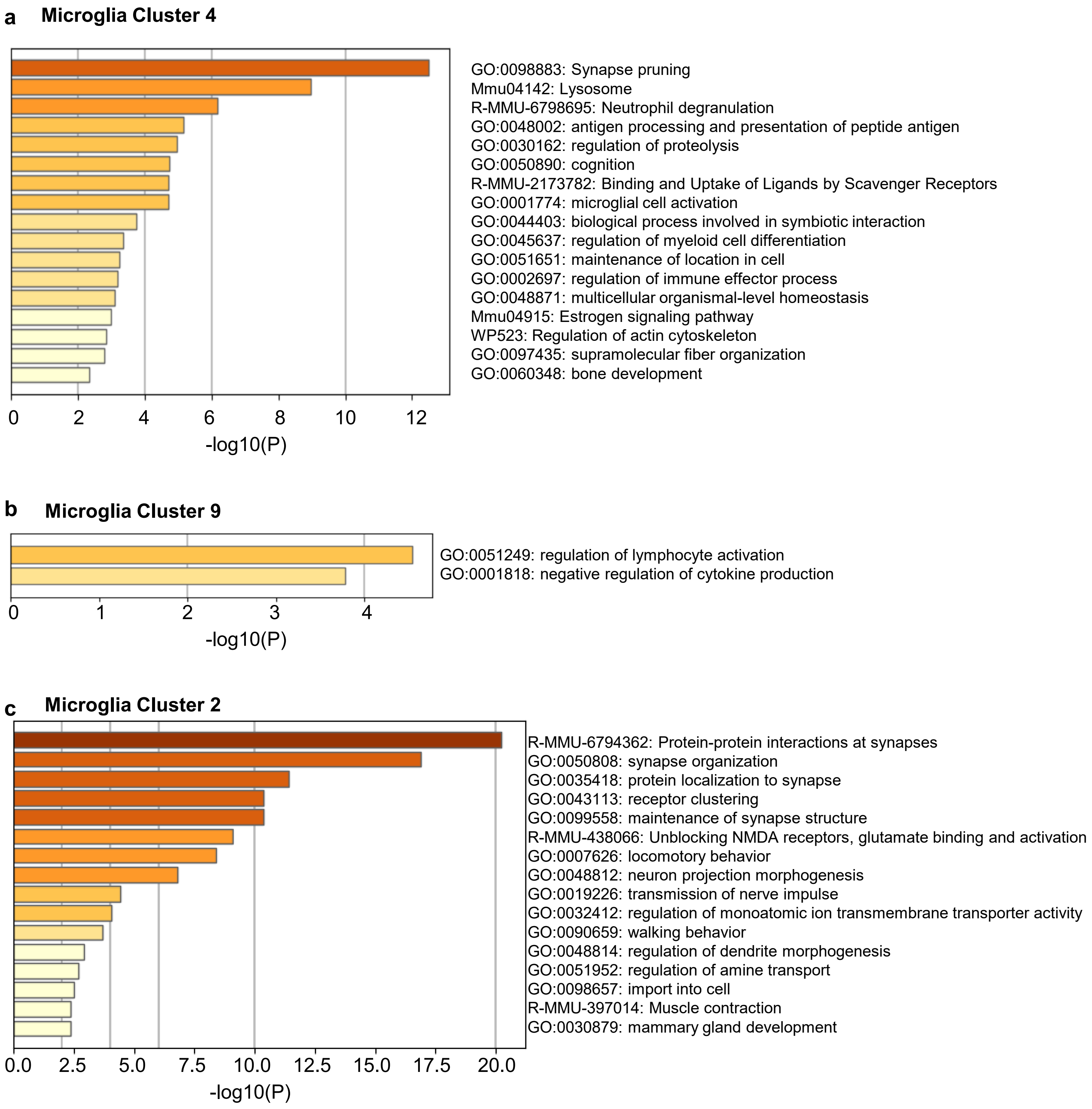

Figure S4: Gene Ontology analysis of microglia clusters: Metascape was used to elucidate Gene Ontology (GO) terms for microglial clusters 4 (**a**), 9 (**b**), and 2 (**c**).

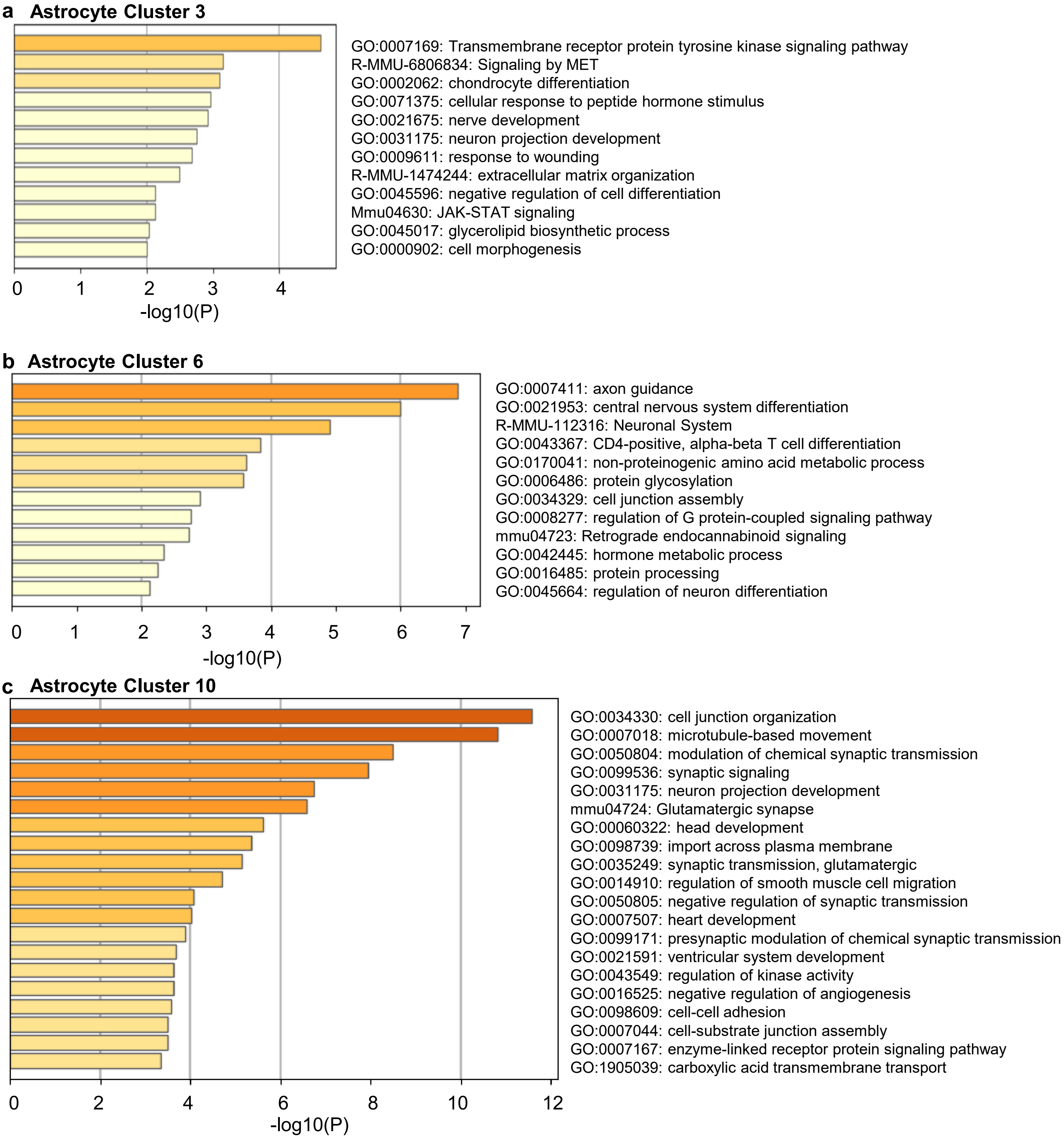

Figure S5: Gene Ontology analysis of astrocyte clusters: Metascape was used to elucidate Gene Ontology (GO) terms for microglial clusters 3 (**a**), 6 (**b**), and 10 (**c**).

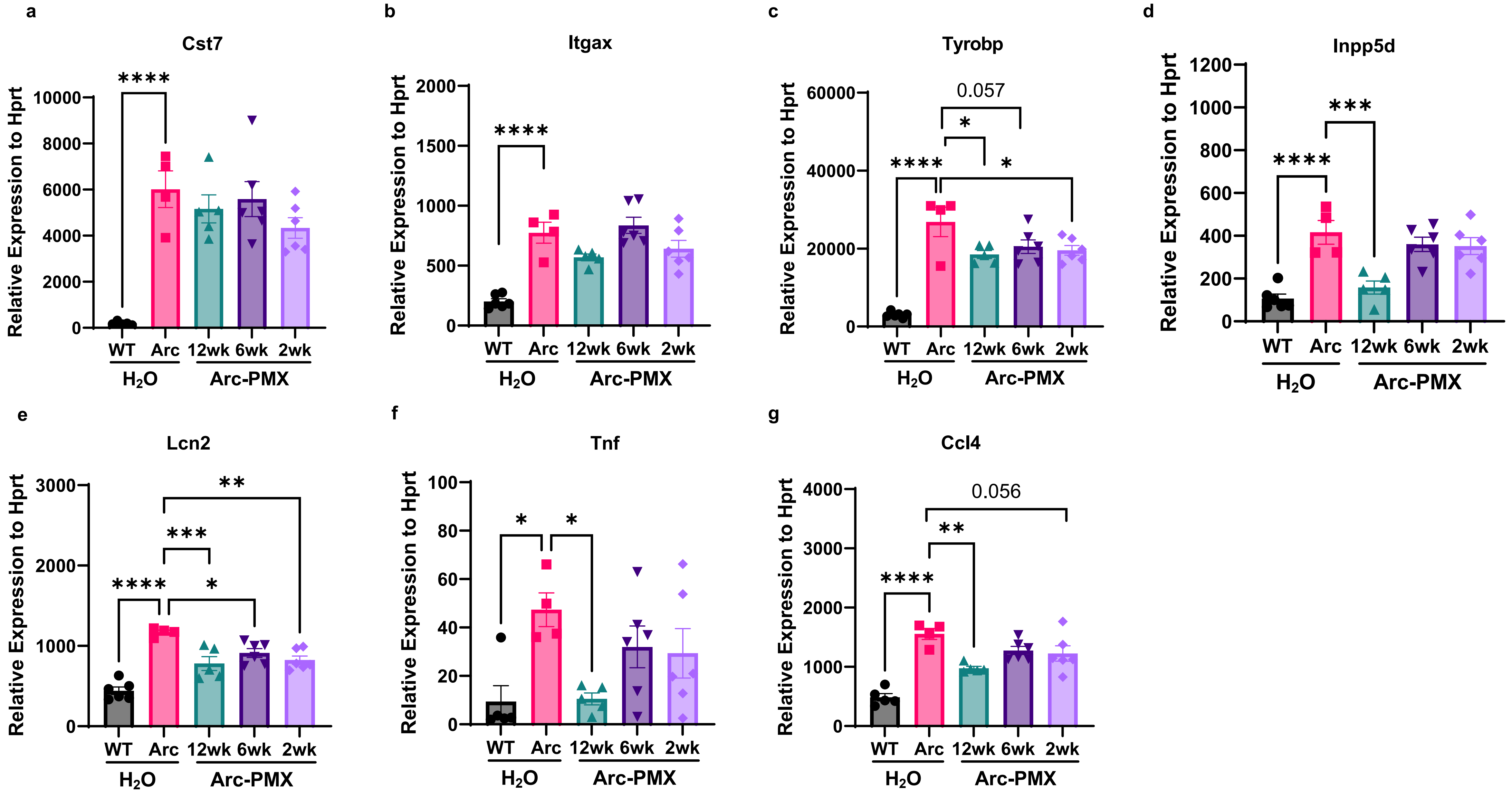

Figure S6: Reduction in inflammatory gene expression with PMX205 treatment in a younger cohort. RNA was extracted from hippocampal homogenates derived from 7-month-old mice treated with PMX205 for different durations and analyzed by qPCR for select reactive microglia genes **(a-d)**, reactive astrocyte gene (**e**), or inflammatory cytokines (**f-g**). Data shown as mean ±SEM. One-way ANOVA with Dunnett's*post hoc*. N = 5 (WT-H_2O), 4 (Arc-H_2O), 5 (Arc-PMX 12wk), 6 (Arc-PMX 6wk), 6 (Arc-PMX 2wk).

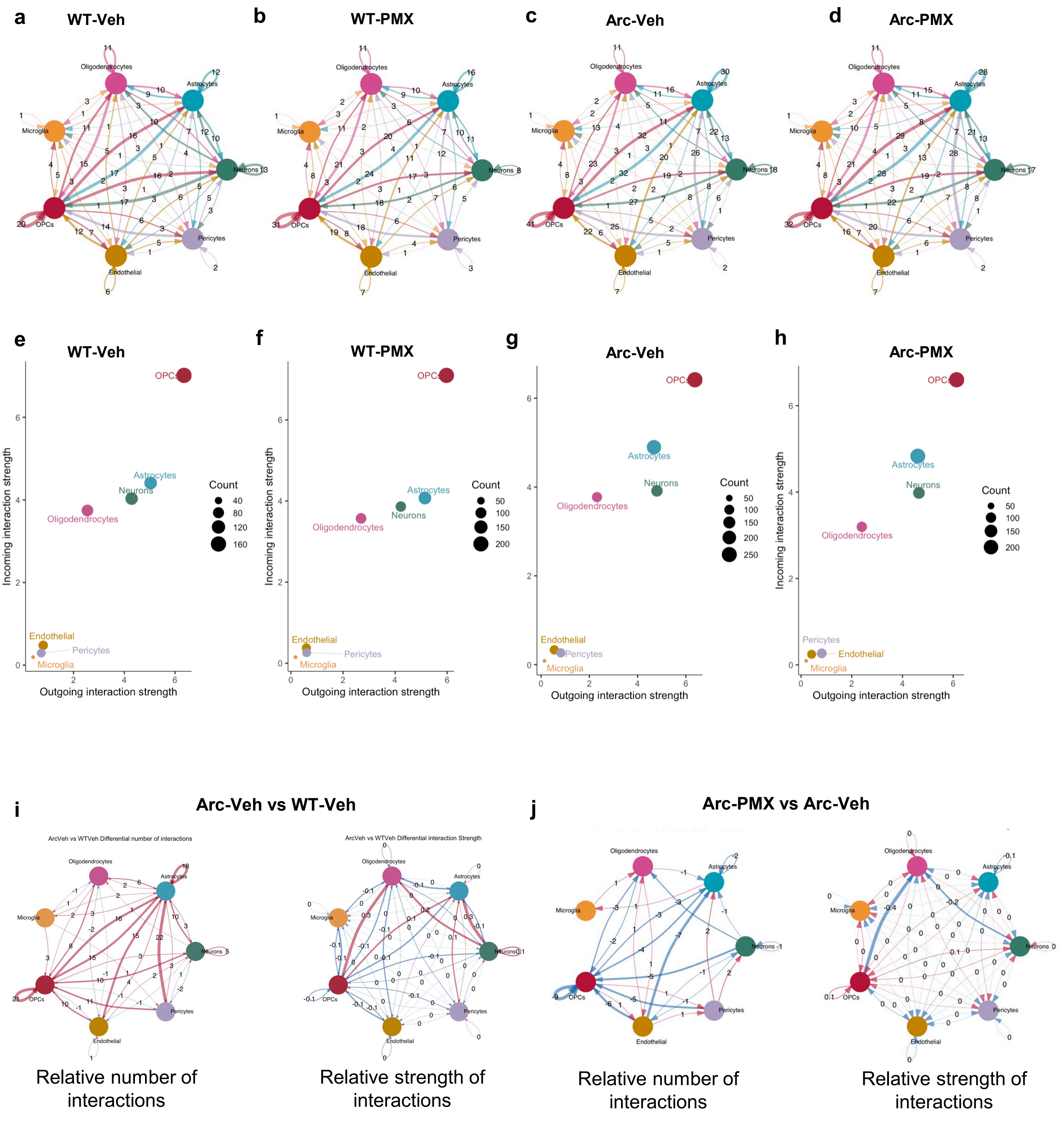

Figure S7: Aberrant intercellular signaling in Arctic hippocampi is suppressed by C5aR1 inhibition. (**a-d**) Aggregated cell-cell communication network hierarchy plot derived with CellChat in WT-Veh (**a**), WT-PMX (**b**), Arc-Veh (**c**), Arc-PMX (**d**). Cell-specific sender and receiver of intercellular signaling shown for WT-Veh (**e**), WT-PMX (**f**), Arc-Veh (**g**), Arc-PMX (**h**) shown as scatter plot made with CellChat. (**i**) Relative number and strength of interactions comparing Arc-veh with WT-veh. (**j**) Relative number and strength of interactions comparing Arc-PMX with Arc-veh. Increased interaction number or strength in Arc-veh (**i**) and Arc-PMX (**j**) are indicated by red lines while decreases are indicated in blue.

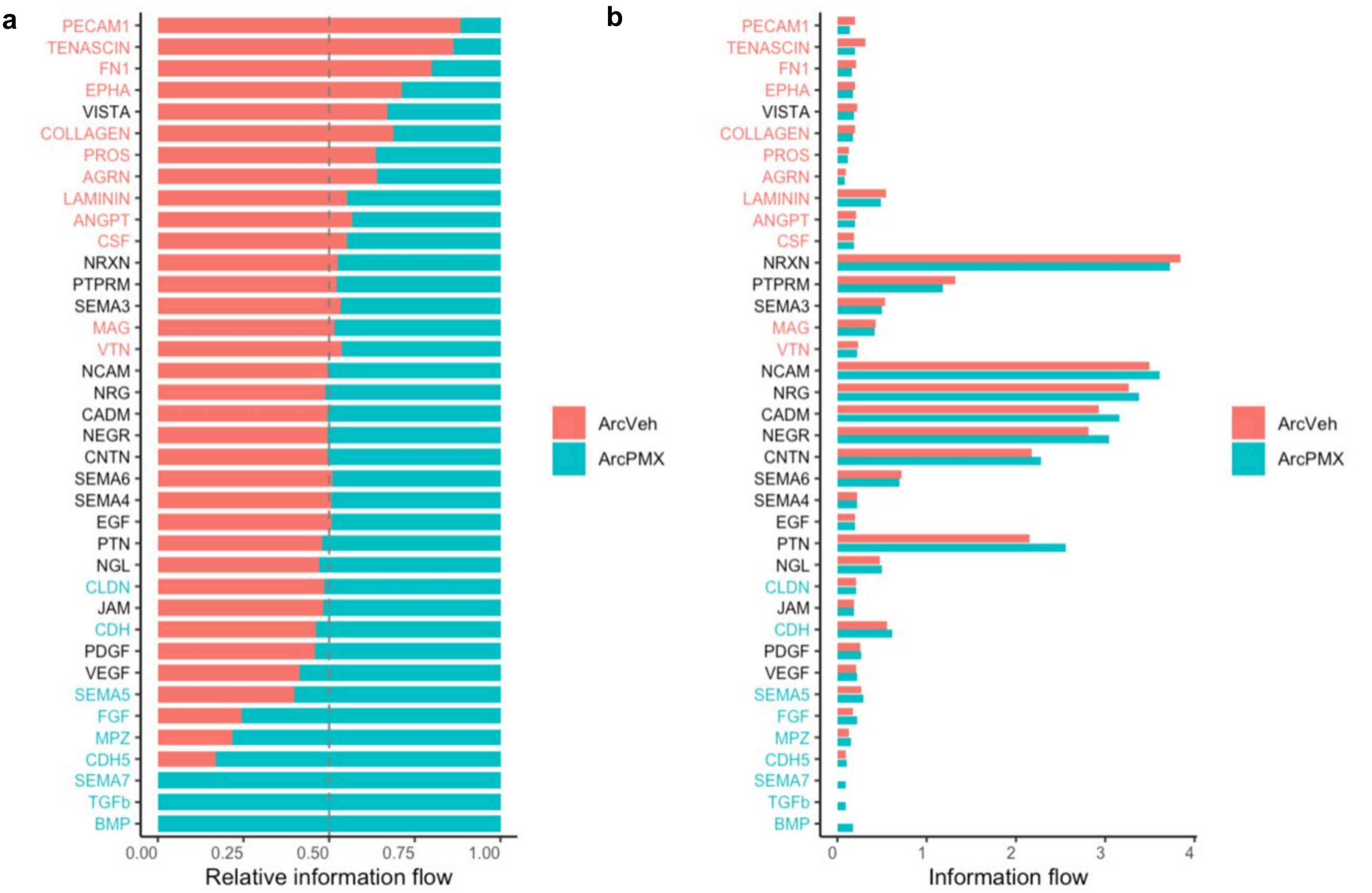

Figure S8: Relative and absolute information flow of pathways enhanced or suppressed by PMX205 treatment in Arctic mice. Total list of cell signaling pathways identified by CellChat in Arc-veh vs Arc-PMX. (**a**) Relative information flow of pathways enhanced by PMX205 treatment (text shown in teal), suppressed by PMX205 (text shown in salmon), or unchanged by treatment (text shown in black). (**b**) Absolute information flow of the pathways identified by CellChat in Arc-Veh and Arc-PMX cells.

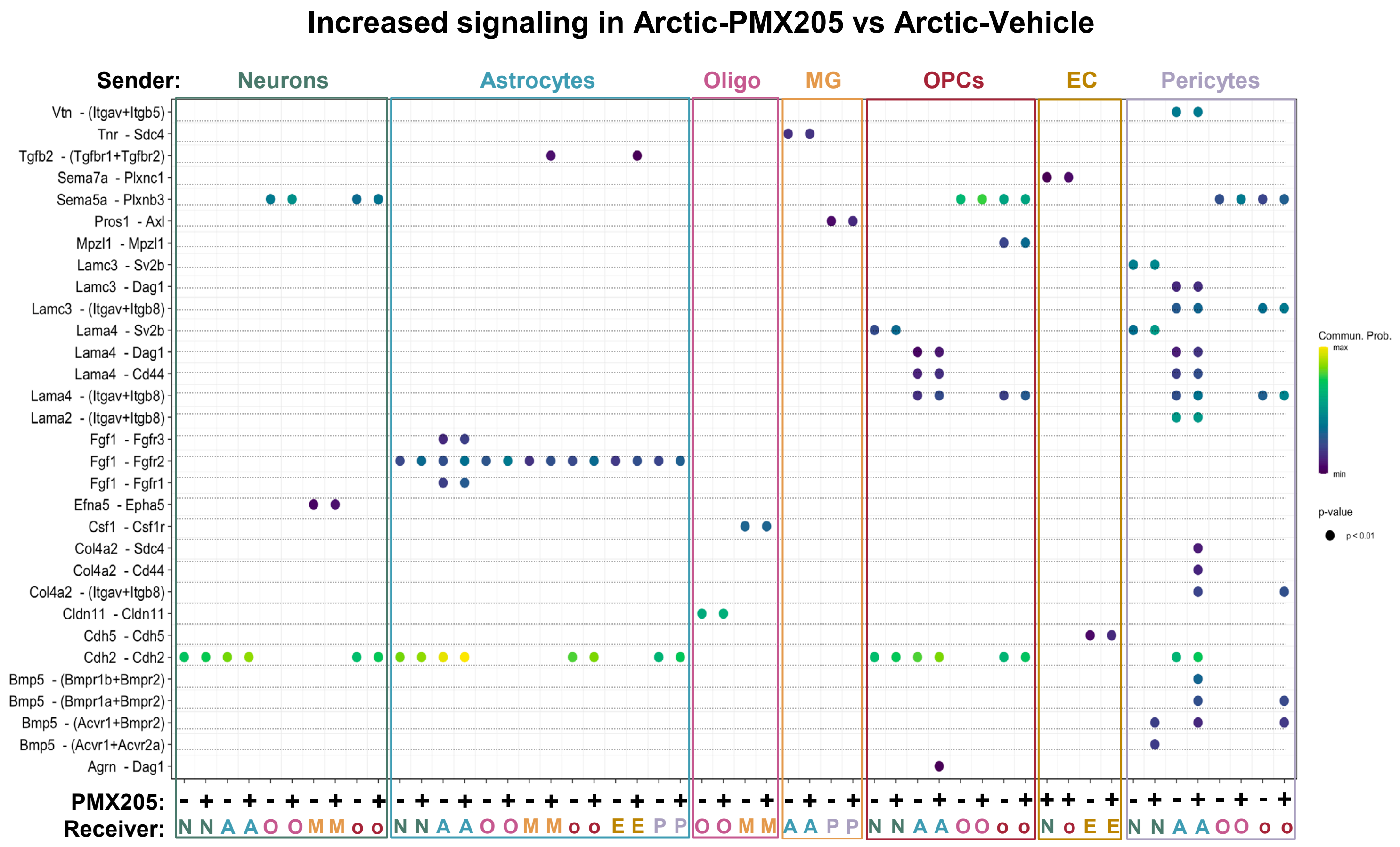

Figure S9: Receptor-Ligand interactions mediated by different cell types in Arc-Veh and Arc-PMX. Signaling probability of receptor-ligand communication, and cellular sender and receiver in pathways that were increased in Arc-PMX compared to Arc-Veh. (Abbreviations: N, Neuron; A, Astrocyte; O, Oligodendrocyte; M, Microglia; o, OPCs; E, Endothelial Cells; P, Pericytes)

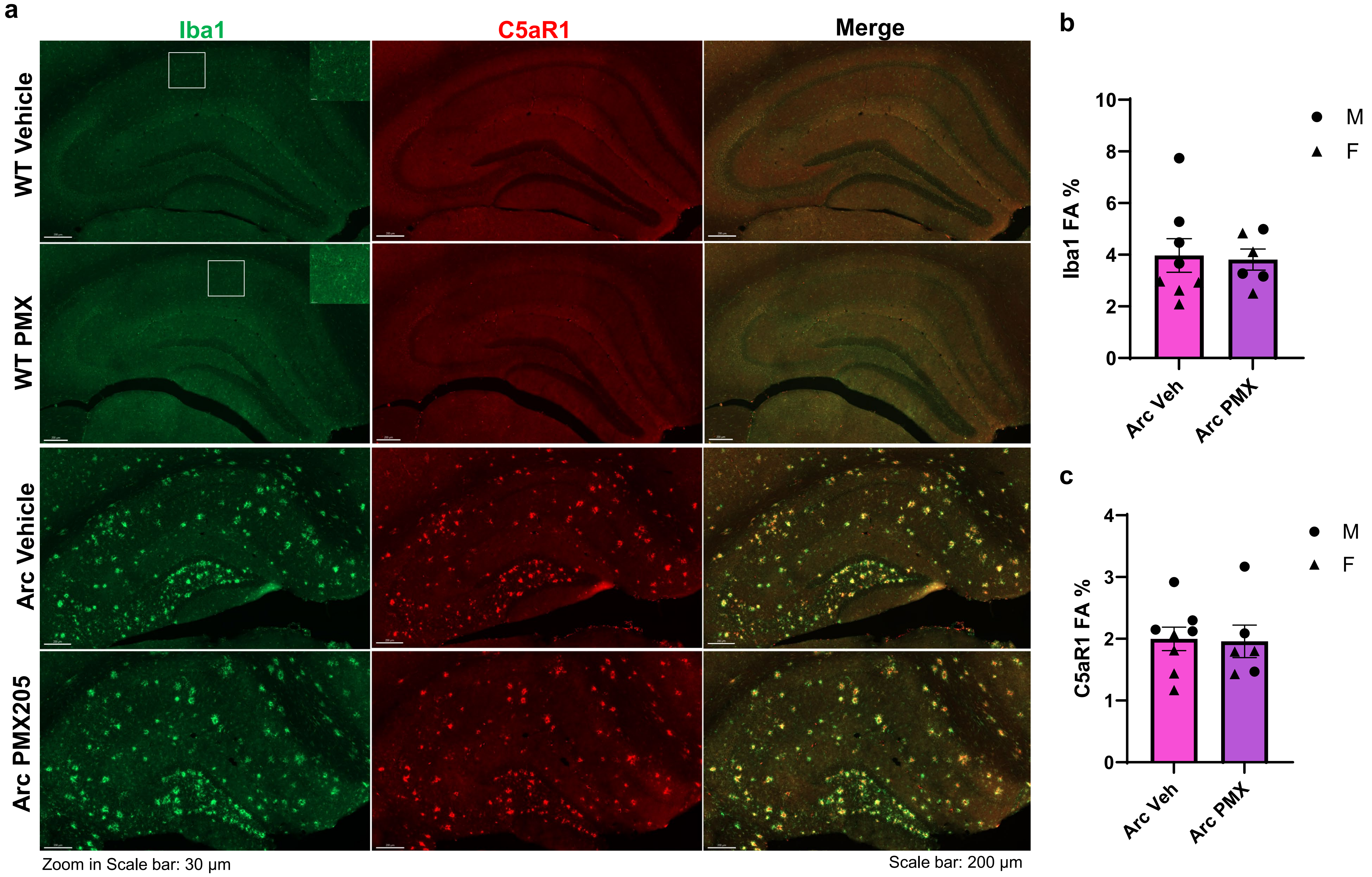

Figure S10: PMX205 did not affect hippocampal C5aR1 protein expression in Arctic mice. (a) Representative images of Iba1 (green) and C5aR1 (red) staining and merged images (far right) in dorsal hippocampal sections derived from mice at 10 months of age. Boxes in the right upper corner of the Iba1 images of WT mice are enlarged from white boxes to show morphology of microglia in WT mice. Quantification of percent field area (FA%) of Iba1 staining **(b)** and C5aR1 staining **(c)** in the hippocampus at 10 months. Data shown as mean ± SEM. N = 6 Arc-Veh, 8 Arc-PMX.

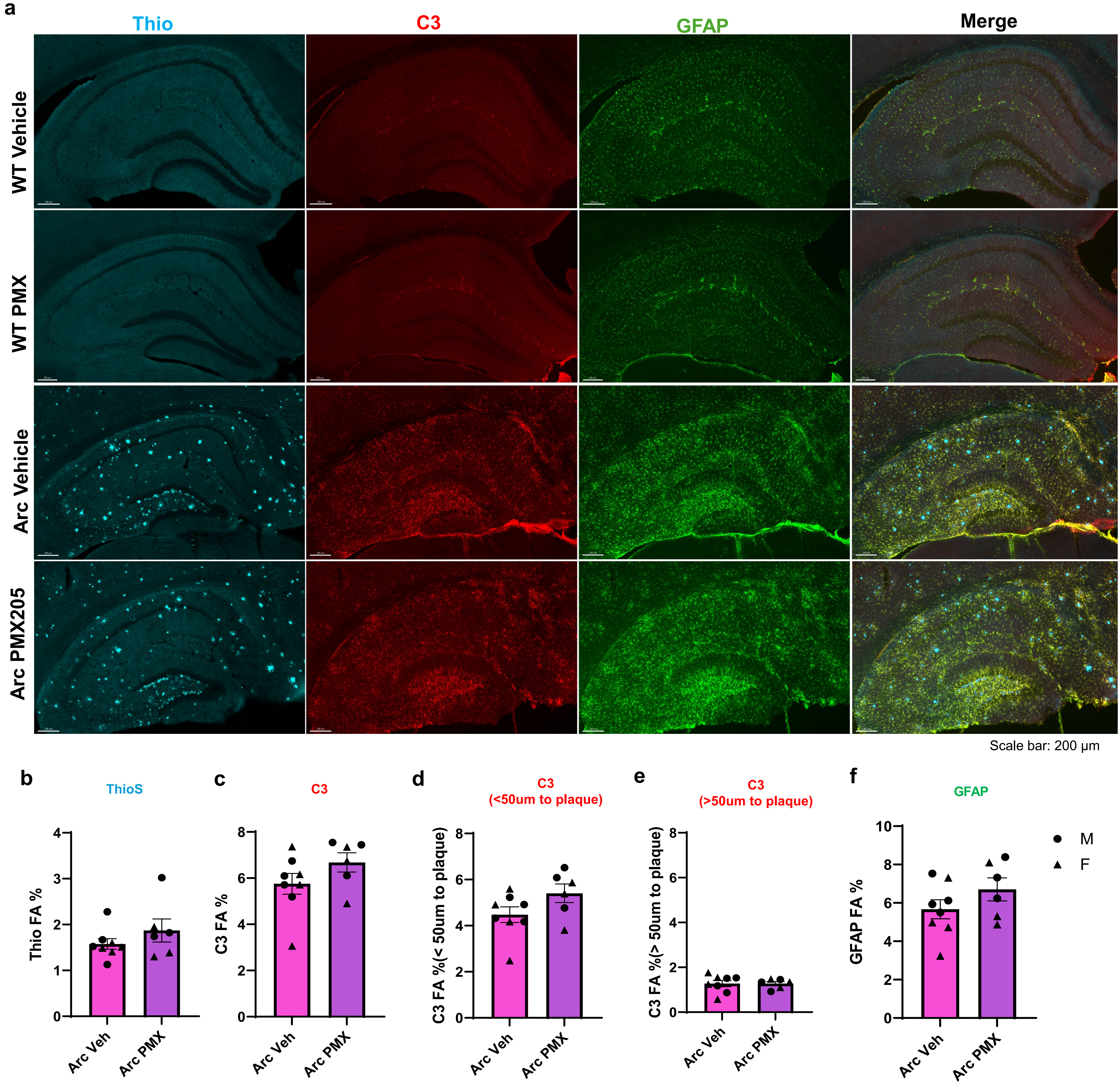

Figure S11: C3 level is elevated around plaque in Arctic and correlated to GFAP level. Representative images of Thioflavine S (pseudo color cyan), C3 (red), GFAP (green) and merged images (far right) in dorsal hippocampal sections derived from 10 months old WT mice with or without PMX205 treatment (top 2 rows) and Arctic mice with or without PMX205 treatment (bottom 2 rows) . Quantification of percent field area of Thioflavine S staining **(b)**, C3 staining **(c-e)** and GFAP staining **(f)** in the hippocampus at 10 months. Data shown as mean ± SEM. N = 6 Arc-Veh, 8 Arc-PMX.

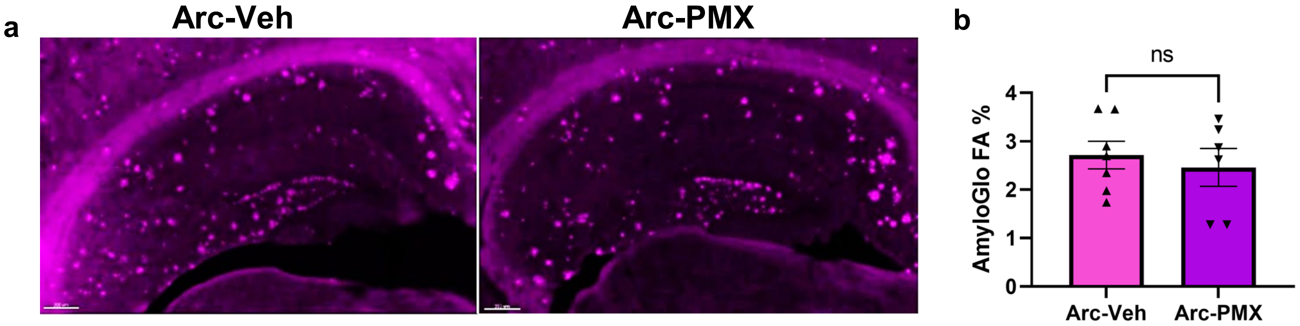

Figure S12: C5aR1 inhibition does not alter hippocampal plaque deposition in Arctic mice. (**a**) Representative images of AmyloGlo staining in dorsal hippocampal sections derived from mice 10 months of age. (**b**) Quantification of percent field area of AmyloGlo staining in the hippocampus. Data shown as mean ± SEM. t-test. N = 7 Arc-veh, 6 Arc-PMX.

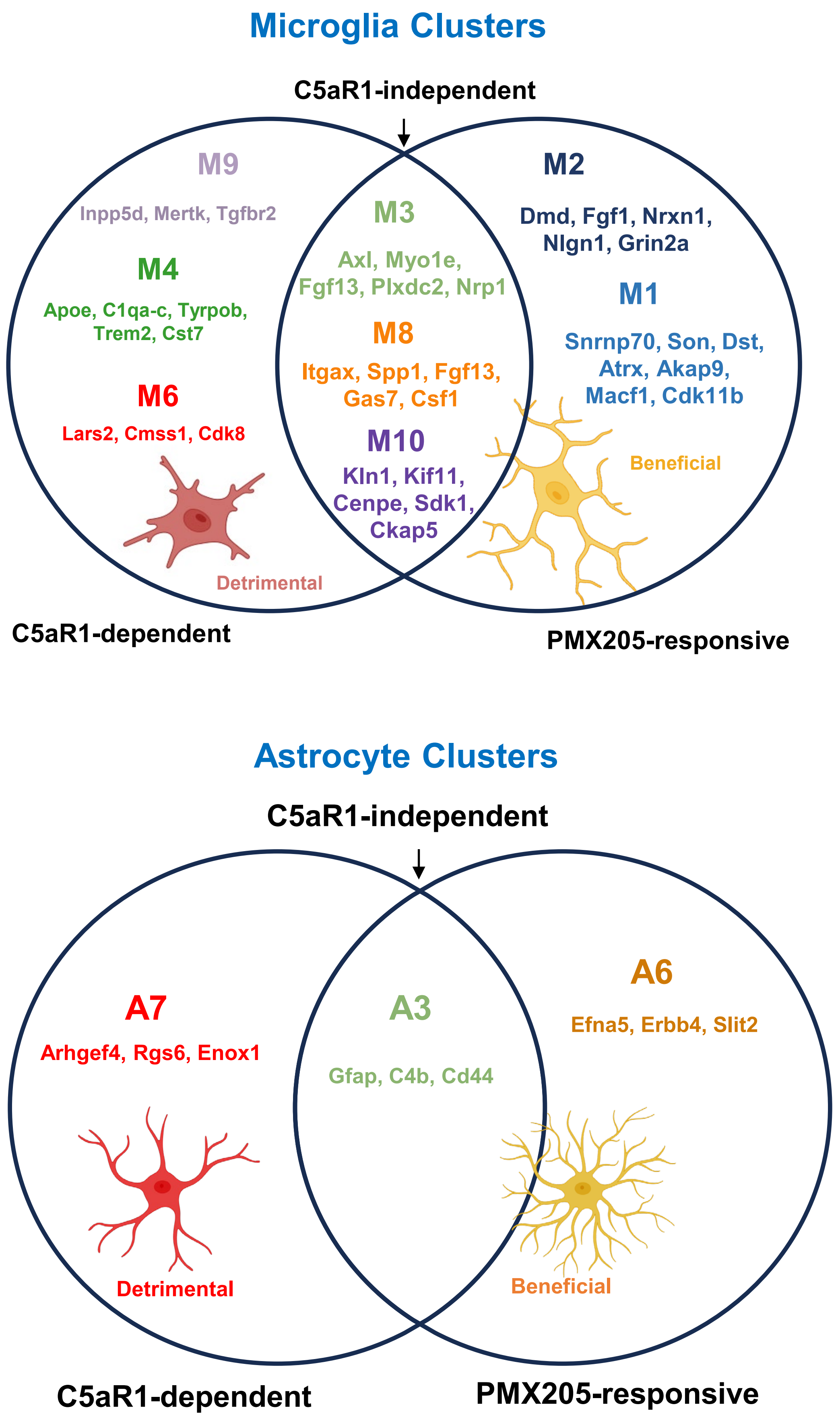

Figure S13: Graphic representation of microglia (top) and astrocyte (bottom) clusters that are affected by PMX205 treatment in Arctic hippocampal cells. Clusters were either upregulated (PMX205-responsive), downregulated (C5aR1-dependent), or unchanged (C5aR1-independent) in response to PMX205 treatment. Figure created with BioRender.com released under a Creative Commons Attribution-NonCommercial-NoDerivs 4.0 International license.
