# Supplementary material for: C5aR1 antagonism suppresses inflammatory glial responses and alters cellular signaling in an Alzheimer’s disease mouse model
Source: Nat Commun. 2024 Aug 15;15:7028. doi: 10.1038/s41467-024-51163-6 (PMC11327341; doi:10.1038/s41467-024-51163-6)
Supplement: Supplementary file 3 — Description of Additional Supplementary Files [file 41467_2024_51163_MOESM3_ESM.pdf]

Description of Additional Supplementary Files

File Name: Supplementary Data 1
Description: Gene List of Clusters shown in Figure 1B

File Name: Supplementary Data 2
Description: Gene list for Microglial Clusters as shown in Figure 3A

File Name: Supplementary Data 3
Description: Gene list for Astrocyte Clusters as shown in Figure 4A
