# Supplementary material for: C5aR1 antagonism suppresses inflammatory glial responses and alters cellular signaling in an Alzheimer’s disease mouse model
Source: Nat Commun. 2024 Aug 15;15:7028. doi: 10.1038/s41467-024-51163-6 (PMC11327341; doi:10.1038/s41467-024-51163-6)
Supplement: Supplementary file 7 — Reporting Summary [file 41467_2024_51163_MOESM7_ESM.pdf]

nature portfolio

Corresponding author(s): Andrea Tenner

Last updated by author(s): Jul 24, 2024

Reporting Summary

Nature Portfolio wishes to improve the reproducibility of the work that we publish. This form provides structure for consistency and transparency in reporting. For further information on Nature Portfolio policies, see our Editorial Policies and the Editorial Policy Checklist.

Statistics

For all statistical analyses, confirm that the following items are present in the figure legend, table legend, main text, or Methods section.

n/a	Confirmed	
☐	☒	The exact sample size (*n*) for each experimental group/condition, given as a discrete number and unit of measurement
☐	☒	A statement on whether measurements were taken from distinct samples or whether the same sample was measured repeatedly
☐	☒	The statistical test(s) used AND whether they are one- or two-sided<br>*Only common tests should be described solely by name; describe more complex techniques in the Methods section.*
☒	☐	A description of all covariates tested
☒	☐	A description of any assumptions or corrections, such as tests of normality and adjustment for multiple comparisons
☐	☒	A full description of the statistical parameters including central tendency (e.g. means) or other basic estimates (e.g. regression coefficient) AND variation (e.g. standard deviation) or associated estimates of uncertainty (e.g. confidence intervals)
☐	☒	For null hypothesis testing, the test statistic (e.g. *F*, *t*, *r*) with confidence intervals, effect sizes, degrees of freedom and *P* value noted<br>*Give P values as exact values whenever suitable.*
☒	☐	For Bayesian analysis, information on the choice of priors and Markov chain Monte Carlo settings
☒	☐	For hierarchical and complex designs, identification of the appropriate level for tests and full reporting of outcomes
☒	☐	Estimates of effect sizes (e.g. Cohen's *d*, Pearson's *r*), indicating how they were calculated

Our web collection on statistics for biologists contains articles on many of the points above.

Software and code

Policy information about availability of computer code

Data collection	No software was used for data collection.
Data analysis	GraphPad Prism V.10.0.2<br>mm10 mus musculus genome assembly (NCBI RefSeq assembly GCF_000001635.20)<br>Fastq files and data matrices were deposited in GEO with the accession ID GSE240950<br>Codes are available on GitHub (https://github.com/heidiiliang/R-code-for-ArcPMX205/tree/main).

For manuscripts utilizing custom algorithms or software that are central to the research but not yet described in published literature, software must be made available to editors and reviewers. We strongly encourage code deposition in a community repository (e.g. GitHub). See the Nature Portfolio guidelines for submitting code & software for further information.

Data

Policy information about availability of data

All manuscripts must include a data availability statement. This statement should provide the following information, where applicable:

- Accession codes, unique identifiers, or web links for publicly available datasets
- A description of any restrictions on data availability
- For clinical datasets or third party data, please ensure that the statement adheres to our policy

Fastq files and data matrices were deposited in GEO with the accession ID GSE240950

Research involving human participants, their data, or biological material

Policy information about studies with human participants or human data. See also policy information about sex, gender (identity/presentation), and sexual orientation and race, ethnicity and racism.

Reporting on sex and gender	*Use the terms sex (biological attribute) and gender (shaped by social and cultural circumstances) carefully in order to avoid confusing both terms. Indicate if findings apply to only one sex or gender; describe whether sex and gender were considered in study design; whether sex and/or gender was determined based on self-reporting or assigned and methods used. Provide in the source data disaggregated sex and gender data, where this information has been collected, and if consent has been obtained for sharing of individual-level data; provide overall numbers in this Reporting Summary. Please state if this information has not been collected. Report sex- and gender-based analyses where performed, justify reasons for lack of sex- and gender-based analysis.*
Reporting on race, ethnicity, or other socially relevant groupings	*Please specify the socially constructed or socially relevant categorization variable(s) used in your manuscript and explain why they were used. Please note that such variables should not be used as proxies for other socially constructed/relevant variables (for example, race or ethnicity should not be used as a proxy for socioeconomic status). Provide clear definitions of the relevant terms used, how they were provided (by the participants/respondents, the researchers, or third parties), and the method(s) used to classify people into the different categories (e.g. self-report, census or administrative data, social media data, etc.) Please provide details about how you controlled for confounding variables in your analyses.*
Population characteristics	*Describe the covariate-relevant population characteristics of the human research participants (e.g. age, genotypic information, past and current diagnosis and treatment categories). If you filled out the behavioural & social sciences study design questions and have nothing to add here, write "See above."*
Recruitment	*Describe how participants were recruited. Outline any potential self-selection bias or other biases that may be present and how these are likely to impact results.*
Ethics oversight	*Identify the organization(s) that approved the study protocol.*

Note that full information on the approval of the study protocol must also be provided in the manuscript.

Field-specific reporting

Please select the one below that is the best fit for your research. If you are not sure, read the appropriate sections before making your selection.

☒ Life sciences ☐ Behavioural & social sciences ☐ Ecological, evolutionary & environmental sciences

For a reference copy of the document with all sections, see nature.com/documents/nr-reporting-summary-flat.pdf

Life sciences study design

All studies must disclose on these points even when the disclosure is negative.

Sample size	Sample size for behavior was determined by a priori power analysis with G*power to determine number of animals per group required to achieve power of 0.8
Data exclusions	Data were not excluded as statistical outliers. One sample was excluded as it was in the WT group but gene expression analysis confirmed that it was Arctic
Replication	Measures were taken to ensure replicability. All experiments were piloted with animals and samples to optimize the methodology. Biological replicates (at least 3) and technical replicates (2-3) were used in assays such as neurofilament light, IHC, and qPCR. All attempts at replication were successful.
Randomization	Mice were randomly assigned to vehicle or PMX205 treatment.
Blinding	Experimenters were blinded to genotype and treatment during behavioural testing and analysis

Reporting for specific materials, systems and methods

We require information from authors about some types of materials, experimental systems and methods used in many studies. Here, indicate whether each material, system or method listed is relevant to your study. If you are not sure if a list item applies to your research, read the appropriate section before selecting a response.

Materials & experimental systems

n/a	Involved in the study
☐	☒ Antibodies
☐	☐ Eukaryotic cell lines
☐	☐ Palaeontology and archaeology
☐	☒ Animals and other organisms
☐	☐ Clinical data
☐	☐ Dual use research of concern
☐	☐ Plants

Methods

n/a	Involved in the study
☐	☐ ChIP-seq
☐	☐ Flow cytometry
☐	☐ MRI-based neuroimaging

Antibodies

Antibodies used

rabbit anti-Iba1 (1:1000, Wako #019-19741), rat anti-CD11b (1:1000, Biorad #MCA74G), hamster anti-CD11c (1:400, Biorad #MCA1369), goat anti-Lcn2 (1:50, R&D #AF1857), chicken anti-GFAP (1:1000, Abcam #ab4674), rabbit anti-S100a6 (1:500, Novus #NBP1-89388), Rat anti-CD88 10/92 (1:1000, BioRad #MCA2456), Rat anti-C3 clone 11H9 (1:50, Hycult #HM1045), and Rabbit anti-GFAP (1:2900, Dako #Z0334). Alexa Fluor secondary antibodies, diluted 1:500 in blocking solution, included goat anti-Armenian hamster alexa 568 (Abcam, # ab175716), goat anti-rabbit alexa 488 (Invitrogen #A11070), goat anti-rat alexa 488 (Invitrogen #A-11006), goat anti-rabbit alexa 555 (Invitrogen #A-21429), goat anti-rat alexa 555 (Invitrogen #A21434), goat anti-rabbit alexa 647 (Invitrogen #A-21244), donkey anti-goat alexa 555 (Invitrogen #A-21432), donkey anti-rabbit alexa 488 (Invitrogen #A-21206), and donkey anti-chicken alexa 647 (Jackson Immuno Research #803605155).

Validation

1. Rabbit anti-Iba1 (1:1000, Wako #019-19741)
a. Host: Rabbit and cross-reactivity species: Human, mouse, and rat. Application: Immunocytochemistry and Immunohistology.
b. Lot #: CAG5175 (Received 2/24/2020) and LEJ1842 (Received 11/3/2021). Certificates and datasheet were filed.
c. https://www.ncbi.nlm.nih.gov/pmc/articles/PMC9277945/
https://www.ncbi.nlm.nih.gov/pmc/articles/PMC8065162/
d. Iba1 IHC images were used in Figure 6A and B.

2. Rat anti-CD11b (1:1000, Biorad #MCA74G)
a. Host: Rat and cross-reactivity species: Mouse, human rabbit. Clone M1/70.15. Application: Flow Cytometry, Immunofluorescence, Immunohistology (frozen and paraffin) and Immunoprecipitation.
b. Batch number: 1804 (Received 5/26/2021) and 156410 (Received 5/28/2021). Specific datasheets by batch # were filed.
c. https://europepmc.org/backend/ptpmcrender.fcgi?accid=PMC6407295&blobtype=pdf
https://europepmc.org/backend/ptpmcrender.fcgi?accid=PMC10029189&blobtype=pdf
d. CD11b IHC images were used in Figure 6A and B.

3. Hamster anti-CD11c (1:400, Biorad #MCA1369)
a. Host: Hamster and cross-reactivity species: Mouse. Application: Western Blot, Immunofluorescence, Immunohistology (frozen and paraffin) and Immunoprecipitation.
b. Lot number 1711 (Received 4/19 and 5/25/2021). Specific datasheet by batch # was filed.
c. https://www.ncbi.nlm.nih.gov/pmc/articles/PMC9277945/
https://actaneurocomms.biomedcentral.com/articles/10.1186/s40478-022-01416-6
d. CD11c IHC images were used in Figure 6A and D.

4. Goat anti-Lcn2 (1:50, R&D #AF1857)
a. Host: Goat and cross-reactivity species: Mouse. Application: Flow Cytometry, Immunofluorescence, Immunohistology (frozen) and Immunoprecipitation.
b. Lot #: JZP0521011 (recid 9/16 and 10/7/2021). Certificates and datasheet were filed.
c. https://jneuroinflammation.biomedcentral.com/counter/pdf/10.1186/s12974-022-02455-5.pdf
https://europepmc.org/backend/ptpmcrender.fcgi?accid=PMC7503651&blobtype=pdf
d. LCN2 IHC images were used in Figure 6E and G.

5. Chicken anti-GFAP (1:1000, Abcam #ab4674)
a. Host: Chicken and cross-reactivity species: Mouse, Rat. Application: Immunofluorescence, Immunohistology (frozen and paraffin) and Western blot.
b. GR33931871(9/15/21 received) and GR3438978-1 (6/2/22 received). Datasheet was filed.
c. https://actaneurocomms.biomedcentral.com/articles/10.1186/s40478-022-01416-6
https://europepmc.org/backend/ptpmcrender.fcgi?accid=PMC7396151&blobtype=pdf
d. GFAP IHC images were used in Figure 6E and H.

6. Rabbit anti-S100a6 (1:500, Novus #NBP1-89388)
a. Host: Rabbit and cross-reactivity species: Human, Mouse, Rat. Application: Immunocytochemistry, Immunofluorescence, Immunohistology (frozen and paraffin) and Western blot.
b. batch #: 000013632 (received 2/20 and 9/30/2022). Certificates and datasheet were filed.
c. https://www.ncbi.nlm.nih.gov/pmc/articles/PMC6675528/

https://www.ncbi.nlm.nih.gov/pmc/articles/PMC8015719/
d. S100a6 IHC images were used in Figure 6E and F.

7. Rat anti-CD88 10/92 (1:1000, BioRad #MCA2456)
a. Host: Rat and cross-reactivity species: Mouse. Application: Immunofluorescence, Immunohistology (frozen) and Flow Cytometry.
b. 152778 (9/2/2021 received). Datasheet was filed.
c. https://www.sciencedirect.com/science/article/pii/S016524780300052X?via%3Dihub
https://www.ncbi.nlm.nih.gov/pmc/articles/PMC2921960/
d. CD88 (C5aR1) IHC images were used in Figure S10A and C.

8. Rat anti-C3 clone 11H9 (1:50, Hycult #HM1045)
a. Host: Rat and cross-reactivity species: Mouse. Application: Immuno assay, Immunofluorescence, Immunohistology (frozen), Western blot and Flow Cytometry.
b. batch #: 35786M0423 (received 3/18/2024). Datasheet was filed.
c. https://www.sciencedirect.com/science/article/pii/S1286457905004314?via%3Dihub
https://www.ncbi.nlm.nih.gov/pmc/articles/PMC2574638/
d. C3 IHC images were used in Figure S11A, C, D and E.

9. Rabbit anti-GFAP (1:2900, Dako #Z0334)
a. Host: Rabbit and cross-reactivity species: Human, cow, mouse, rat, and dog. Application: Immunofluorescence, Immunohistology (frozen and paraffin) and Western blot.
b. 20044021(5/10/17 received). Datasheet was filed.
c. https://europepmc.org/backend/ptpmcrender.fcgi?accid=PMC6174950&blobtype=pdf
https://www.mdpi.com/1422-0067/22/6/3233
d. GFAP IHC images were used in Figure S11A and F.

Eukaryotic cell lines

Policy information about cell lines and Sex and Gender in Research

Cell line source(s): *State the source of each cell line used and the sex of all primary cell lines and cells derived from human participants or vertebrate models.*

Authentication: *Describe the authentication procedures for each cell line used OR declare that none of the cell lines used were authenticated.*

Mycoplasma contamination: *Confirm that all cell lines tested negative for mycoplasma contamination OR describe the results of the testing for mycoplasma contamination OR declare that the cell lines were not tested for mycoplasma contamination.*

Commonly misidentified lines (See ICLAC register): *Name any commonly misidentified cell lines used in the study and provide a rationale for their use.*

Palaeontology and Archaeology

Specimen provenance: *Provide provenance information for specimens and describe permits that were obtained for the work (including the name of the issuing authority, the date of issue, and any identifying information). Permits should encompass collection and, where applicable, export.*

Specimen deposition: *Indicate where the specimens have been deposited to permit free access by other researchers.*

Dating methods: *If new dates are provided, describe how they were obtained (e.g. collection, storage, sample pretreatment and measurement), where they were obtained (i.e. lab name), the calibration program and the protocol for quality assurance OR state that no new dates are provided.*

☐ Tick this box to confirm that the raw and calibrated dates are available in the paper or in Supplementary Information.

Ethics oversight: *Identify the organization(s) that approved or provided guidance on the study protocol, OR state that no ethical approval or guidance was required and explain why not.*

Note that full information on the approval of the study protocol must also be provided in the manuscript.

Animals and other research organisms

Policy information about studies involving animals; ARRIVE guidelines recommended for reporting animal research, and Sex and Gender in Research

Laboratory animals: Arctic48 mouse model of AD which carries the human APP transgene with three mutations – the Indiana (V717F), the Swedish (K670N + M671L), and the Arctic (E22G), was generated on a C57BL6/J background and originally provided by Dr. Lennart Mucke

	(Gladstone Institute, San Francisco, CA). Mice were between the ages of 4 months and 10 months during experimental timeline. Mice were single-housed at ambient temperature/humidity for the duration of treatment with free access to food and water.
Wild animals	None used.
Reporting on sex	Both males and females were used in this study. Results were compared on the basis of sex and where no differences were found, the sexes were analyzed as one group
Field-collected samples	None
Ethics oversight	The Institutional Animal Care and Use Committee of University of California at Irvine granted ethical approved all animal procedures (#AUP-21-132). Experiments were performed according to the NIH Guide for the Care and Use of laboratory animals.

Note that full information on the approval of the study protocol must also be provided in the manuscript.

Clinical data

Policy information about clinical studies

All manuscripts should comply with the ICMJE guidelines for publication of clinical research and a completed CONSORT checklist must be included with all submissions.

Clinical trial registration	*Provide the trial registration number from ClinicalTrials.gov or an equivalent agency.*
Study protocol	*Note where the full trial protocol can be accessed OR if not available, explain why.*
Data collection	*Describe the settings and locales of data collection, noting the time periods of recruitment and data collection.*
Outcomes	*Describe how you pre-defined primary and secondary outcome measures and how you assessed these measures.*

Dual use research of concern

Policy information about dual use research of concern

Hazards

Could the accidental, deliberate or reckless misuse of agents or technologies generated in the work, or the application of information presented in the manuscript, pose a threat to:

No	Yes	
☒	☐	Public health
☒	☐	National security
☒	☐	Crops and/or livestock
☒	☐	Ecosystems
☒	☐	Any other significant area

Experiments of concern

Does the work involve any of these experiments of concern:

No	Yes	
☒	☐	Demonstrate how to render a vaccine ineffective
☒	☐	Confer resistance to therapeutically useful antibiotics or antiviral agents
☒	☐	Enhance the virulence of a pathogen or render a nonpathogen virulent
☒	☐	Increase transmissibility of a pathogen
☒	☐	Alter the host range of a pathogen
☒	☐	Enable evasion of diagnostic/detection modalities
☒	☐	Enable the weaponization of a biological agent or toxin
☒	☐	Any other potentially harmful combination of experiments and agents

Plants

Seed stocks	*Report on the source of all seed stocks or other plant material used. If applicable, state the seed stock centre and catalogue number. If plant specimens were collected from the field, describe the collection location, date and sampling procedures.*

Novel plant genotypes

Describe the methods by which all novel plant genotypes were produced. This includes those generated by transgenic approaches, gene editing, chemical/radiation-based mutagenesis and hybridization. For transgenic lines, describe the transformation method, the number of independent lines analyzed and the generation upon which experiments were performed. For gene-edited lines, describe the editor used, the endogenous sequence targeted for editing, the targeting guide RNA sequence (if applicable) and how the editor was applied.

Authentication

Describe any authentication procedures for each seed stock used or novel genotype generated. Describe any experiments used to assess the effect of a mutation and, where applicable, how potential secondary effects (e.g. second site T-DNA insertions, mosiacism, off-target gene editing) were examined.

ChIP-seq

Data deposition

☐ Confirm that both raw and final processed data have been deposited in a public database such as GEO.

☐ Confirm that you have deposited or provided access to graph files (e.g. BED files) for the called peaks.

Data access links
May remain private before publication.

For "Initial submission" or "Revised version" documents, provide reviewer access links. For your "Final submission" document, provide a link to the deposited data.

Files in database submission

Provide a list of all files available in the database submission.

Genome browser session
(e.g. UCSC)

Provide a link to an anonymized genome browser session for "Initial submission" and "Revised version" documents only, to enable peer review. Write "no longer applicable" for "Final submission" documents.

Methodology

Replicates

Describe the experimental replicates, specifying number, type and replicate agreement.

Sequencing depth

Describe the sequencing depth for each experiment, providing the total number of reads, uniquely mapped reads, length of reads and whether they were paired- or single-end.

Antibodies

Describe the antibodies used for the ChIP-seq experiments; as applicable, provide supplier name, catalog number, clone name, and lot number.

Peak calling parameters

Specify the command line program and parameters used for read mapping and peak calling, including the ChIP, control and index files used.

Data quality

Describe the methods used to ensure data quality in full detail, including how many peaks are at FDR 5% and above 5-fold enrichment.

Software

Describe the software used to collect and analyze the ChIP-seq data. For custom code that has been deposited into a community repository, provide accession details.

Flow Cytometry

Plots

Confirm that:

☐ The axis labels state the marker and fluorochrome used (e.g. CD4-FITC).

☐ The axis scales are clearly visible. Include numbers along axes only for bottom left plot of group (a 'group' is an analysis of identical markers).

☐ All plots are contour plots with outliers or pseudocolor plots.

☐ A numerical value for number of cells or percentage (with statistics) is provided.

Methodology

Sample preparation

Describe the sample preparation, detailing the biological source of the cells and any tissue processing steps used.

Instrument

Identify the instrument used for data collection, specifying make and model number.

Software

Describe the software used to collect and analyze the flow cytometry data. For custom code that has been deposited into a community repository, provide accession details.

Cell population abundance

Describe the abundance of the relevant cell populations within post-sort fractions, providing details on the purity of the samples and how it was determined.

Gating strategy

Describe the gating strategy used for all relevant experiments, specifying the preliminary FSC/SSC gates of the starting cell population, indicating where boundaries between "positive" and "negative" staining cell populations are defined.

☐ Tick this box to confirm that a figure exemplifying the gating strategy is provided in the Supplementary Information.

Magnetic resonance imaging

Experimental design

Design type	*Indicate task or resting state; event-related or block design.*
Design specifications	*Specify the number of blocks, trials or experimental units per session and/or subject, and specify the length of each trial or block (if trials are blocked) and interval between trials.*
Behavioral performance measures	*State number and/or type of variables recorded (e.g. correct button press, response time) and what statistics were used to establish that the subjects were performing the task as expected (e.g. mean, range, and/or standard deviation across subjects).*

Acquisition

Imaging type(s)	*Specify: functional, structural, diffusion, perfusion.*
Field strength	*Specify in Tesla*
Sequence & imaging parameters	*Specify the pulse sequence type (gradient echo, spin echo, etc.), imaging type (EPI, spiral, etc.), field of view, matrix size, slice thickness, orientation and TE/TR/flip angle.*
Area of acquisition	*State whether a whole brain scan was used OR define the area of acquisition, describing how the region was determined.*

Diffusion MRI ☐ Used ☐ Not used

Preprocessing

Preprocessing software	*Provide detail on software version and revision number and on specific parameters (model/functions, brain extraction, segmentation, smoothing kernel size, etc.).*
Normalization	*If data were normalized/standardized, describe the approach(es): specify linear or non-linear and define image types used for transformation OR indicate that data were not normalized and explain rationale for lack of normalization.*
Normalization template	*Describe the template used for normalization/transformation, specifying subject space or group standardized space (e.g. original Talairach, MNI305, ICBM152) OR indicate that the data were not normalized.*
Noise and artifact removal	*Describe your procedure(s) for artifact and structured noise removal, specifying motion parameters, tissue signals and physiological signals (heart rate, respiration).*
Volume censoring	*Define your software and/or method and criteria for volume censoring, and state the extent of such censoring.*

Statistical modeling & inference

Model type and settings	*Specify type (mass univariate, multivariate, RSA, predictive, etc.) and describe essential details of the model at the first and second levels (e.g. fixed, random or mixed effects; drift or auto-correlation).*
Effect(s) tested	*Define precise effect in terms of the task or stimulus conditions instead of psychological concepts and indicate whether ANOVA or factorial designs were used.*

Specify type of analysis: ☐ Whole brain ☐ ROI-based ☐ Both

Statistic type for inference<br>(See Eklund et al. 2016)	*Specify voxel-wise or cluster-wise and report all relevant parameters for cluster-wise methods.*
Correction	*Describe the type of correction and how it is obtained for multiple comparisons (e.g. FWE, FDR, permutation or Monte Carlo).*

Models & analysis

n/a	Involved in the study
☐	☐ Functional and/or effective connectivity
☐	☐ Graph analysis
☐	☐ Multivariate modeling or predictive analysis

Functional and/or effective connectivity	*Report the measures of dependence used and the model details (e.g. Pearson correlation, partial correlation, mutual information).*
Graph analysis	*Report the dependent variable and connectivity measure, specifying weighted graph or binarized graph, subject- or group-level, and the global and/or node summaries used (e.g. clustering coefficient, efficiency, etc.).*
Multivariate modeling and predictive analysis	*Specify independent variables, features extraction and dimension reduction, model, training and evaluation metrics.*
